# Alleviation of Heavy Metal Stress in Plants and Remediation of Soil by Rhizosphere Microorganisms

**DOI:** 10.3389/fmicb.2017.01706

**Published:** 2017-09-06

**Authors:** Jitendra Mishra, Rachna Singh, Naveen K. Arora

**Affiliations:** Rhizosphere Microbiology Laboratory, Department of Environmental Microbiology, Babasaheb Bhimrao Ambedkar University Lucknow, India

**Keywords:** heavy metal, metal–microbe interaction, bioremediation, phytoremediation, contaminated soils

## Abstract

Increasing concentration of heavy metals (HM) due to various anthropogenic activities is a serious problem. Plants are very much affected by HM pollution particularly in contaminated soils. Survival of plants becomes tough and its overall health under HM stress is impaired. Remediation of HM in contaminated soil is done by physical and chemical processes which are costly, time-consuming, and non-sustainable. Metal–microbe interaction is an emerging but under-utilized technology that can be exploited to reduce HM stress in plants. Several rhizosphere microorganisms are known to play essential role in the management of HM stresses in plants. They can accumulate, transform, or detoxify HM. In general, the benefit from these microbes can have a vast impact on plant’s health. Plant–microbe associations targeting HM stress may provide another dimension to existing phytoremediation and rhizoremediation uses. In this review, applied aspects and mechanisms of action of heavy metal tolerant-plant growth promoting (HMT-PGP) microbes in ensuring plant survival and growth in contaminated soils are discussed. The use of HMT-PGP microbes and their interaction with plants in remediation of contaminated soil can be the approach for the future. This low input and sustainable biotechnology can be of immense use/importance in reclaiming the HM contaminated soils, thus increasing the quality and yield of such soils.

## Introduction

Heavy metals (HM) are metals of high density. Regardless of debate on their classification, the term HM particularly in biological sense is more often used for those metals and semimetals with potential human or environmental toxicity (Tchounwou et al., 2012). Although soils are natural source of HM, geologic and anthropogenic activities increase their concentration to levels that are harmful to both plants and animals (Chibuike and Obiora, 2014). HM can be transported over long distances in gaseous as well as particulate phases (Adriano et al., 2005) which leads to their rapid accumulation in soil, water, and living systems. Although certain HM are essential for optimum plant growth but excessive amounts are harmful to the plants and other organisms in the food chain. Activities such as unpreceded use of agrochemicals and long-term application of urban sewage sludge, industrial waste disposal, waste incineration, and vehicle exhausts are the main sources of HM in agricultural soils. Soil with high concentrations of HM lead to their absorption and accumulation by plant, which ultimately pass into humans via food chain (Zhuang et al., 2013, 2014). Both underground and aboveground surfaces of plants can absorb HM which directly or indirectly affect plant health (Patra et al., 2004). Direct consequences are inhibition of cytoplasmic enzymes and damage to cell structures due to oxidative stress (Jadia and Fulekar, 2009). Oxidative stress is related to formation of reactive oxygen species (ROS) and cytotoxic compounds like methylglyoxal (MG) and perturbs the equilibrium of ionic homeostasis within the plant cells (Hossain et al., 2012; Sytar et al., 2013). Some HM indirectly impose oxidative stress via multiple mechanisms including glutathione depletion, binding to sulfhydryl groups of proteins (Valko et al., 2005), inhibiting antioxidative enzymes, or inducing ROS-producing enzymes like NADPH oxidases (Bielen et al., 2013). Whether direct or indirect, plants exposed to high levels of HM result in reduction or even complete cessation of all metabolic activities. Although it has been known that plants possess several defense strategies to avoid or tolerate HM intoxication but beyond certain limits these mechanisms fail and survival of plant is jeopardized (Clemens and Ma, 2016). Hence, it becomes very essential to remove the accumulated HM for normal functioning of plant and also protect organisms dependent on them. The techniques being used for HM cleanup from contaminated sites include excavation (physical removal from contaminated sites), stabilization or *in situ* fixation (stabilization by adding chemicals to alter metal to a state that is not absorbed by plants), and soil washing (reduction of HM by physical or chemical extraction). However, these physical processes are neither efficient nor cost effective (Schnoor, 1997). Therefore, the quest for cost effective, durable, and environmental friendly solutions to cleanup HM should be on priority. In recent past several biological means have been considered (Gavrilescu, 2004; Wuana and Okieimen, 2011). In this context phytoremediation (the use of growing plants reduces the concentration of HM in the soil) and use of rhizospheric microbes have emerged as important alternatives to ensure high efficiency and better performance. Rhizospheric microbes in particular show abilities to protect the plant from HM stress as well as help in their accumulation from soil. Microbes have metabolic capabilities supported by molecular machinery to adapt and perform even in presence of high concentration of HM. This review is focused on current understanding of rhizospheric microbes in relation to remediation of HM contamination. The review also discusses the utilization of rhizospheric microbes in fighting the HM stress in plants.

## HM and Rhizospheric Microbes

In terrestrial ecosystems, soils are the major sink for metal contamination (Gadd, 2010). Metal concentration may range in typical soil from 1 to 100,000 mg/kg (Long et al., 2002) of which a significant part is transformed by geo-active action of soil microbes. Soil microbes especially the rhizospheric population play important role in HM detoxification in contaminated soils. This input of the rhizomicrobial population is also referred to as rhizoremediation (Kuiper et al., 2004). This involves higher metabolic activity of microbes including prokaryotes and eukaryotes near the vicinity of plants’ root. According to Pires et al. (2017) the bacterial population in HM contaminated sites is predominantly composed of Firmicutes, Proteobacteria, and Actinobacteria and most represented genera belong to *Bacillus*, *Pseudomonas*, and *Arthrobacter*. Rhizobia are also very important plant growth promoting (PGP) microbes found in the rhizosphere. In fact nodulation and nitrogenase activities can be very sensitive to HM stress but HMT rhizobial strains have also been reported from contaminated sites effectively carrying out symbiotic nitrogen fixation. Legume–rhizobia symbiosis is widely known to detoxify HM and improves the quality of contaminated soils (Checcucci et al., 2017). In case of fungi, Ascomycota and Basidiomycota are the most commonly reported from HM contaminated soils (Narendrula-Kotha and Nkongolo, 2017). However, it has been also observed that nutrient poor but HM contaminated soils are often primarily colonized by arbuscular mycorrhizal (AM) fungi (Khan et al., 2000). High load of HM in these contaminated soils is not a problem for them. In fact, various intracellular functions of AM fungi and other rhizosphere microbes are driven by binding metal ions present in the external environment on the cell surface or to transport them into the cell (Ehrlich, 1997). In soil, they can change the metal speciation, toxicity, mobility, dissolution, and deterioration (Gadd, 2010). A large number of metals can be transformed by these microbes. Metal microbes interaction in rhizosphere is very stringent and somehow depends upon physico-chemical nature of soil, type and concentration of metal species, metabolic activity, and diversity of microbes. For further information on metal–microbes interaction one can see reviews by Khan (2005), Giller et al. (2009), Gadd (2010), and Kong and Glick (2017).

## Mechanisms of HM Remediation by HMT-PGP Microbes

**FIGURE 1:**
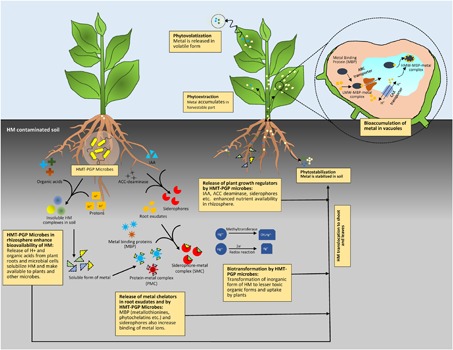
Mechanisms involved in remediation of HM contaminated soil by HMT-PGP microbes–plant interaction.

Alleviation of HM in soil largely depends upon their availability. However, bioavailability of HM can further impair the process of phytoremediation as HM toxicity causes inefficient plant growth. HMT-PGP microbes in the rhizosphere tackle these two major problems simultaneously by modulating plant growth as well as by altering physico-chemical properties of soil to enhance metal bioavailability which trigger rapid detoxification or removal of HM from soil. **Figure 1** provides schematic representation of diverse mechanisms involved in detoxification and remediation of HM in contaminated soils.

HMT-PGP microbes alter metal bioavailability in soil through acidification, chelation, complexation, precipitation, and redox reactions. Acidic pH conditions favor bioavailability and adsorption of HM in rhizosphere (Merdy et al., 2009). Organic acids released by HMT-PGP microbes lower soil pH and sequester soluble metal ions (Turnau and Kottke, 2005). Experimental evidences suggest that a wide array of bacteria and fungi produce organic acids as natural chelating agents of HM (Seneviratne et al., 2017). Gluconic, oxalic, acetic, and malic acids are mainly reported for HM solubilization by soil microbes (Ullah et al., 2015; Gube, 2016). In a study, Fomina et al. (2005) showed that over secretion of organic acids (oxalic and citric) by HM tolerant *Beauveria caledonica* solubilized Cd, Cu, Pb, and Zn metals. The oxalate crystals produced by mycorrhizal fungi are also known to immobilize and detoxify HM (Gadd et al., 2014). Their filamentous hyphal structure deeply penetrates in to the deeper soil aggregates and chelates or adsorbs HM. A study by Kaewdoung et al. (2016) involving scanning electron microscopy equipped with energy dispersive X-ray microanalysis (SEM-EDXA) and X-ray powder diffraction (XRPD) revealed that oxalate crystals produced by wood-rotting fungi *Fomitopsis cf. meliae* and *Ganoderma aff. steyaertanum* contributed in metal tolerance by transforming the metals into less toxic forms (zinc sulfate into zinc oxalate dihydrate, copper sulfate into copper oxalate hydrate, cadmium sulfate into cadmium oxalate trihydrate, and lead nitrate into lead oxalate).

Root exudates also play important role in changing metal bioavailability, as release of certain organic compounds not only mobilizes metals by forming metal complexes but also provide nutrient and energy sources to microbial communities which in turn support plant growth and survival. Root exudates contain organic acids, amino acids, and phytochelatins (PC) which perform as intracellular binding compounds for HM. Release of protons (H^+^) and enzymes with root exudates helps in acidification and electron transfer in the rhizosphere which leads to enhanced metal bioavailability (Ma et al., 2016). Changes in concentrations of exudate compounds in the presence of particular HM can also help in developing biomarkers. Recently, based on gas chromatography-mass spectrometry (GC-MS) and metabolomics methods, Luo et al. (2017) showed that Pb-accumulating and *Sedum alfredii* can significantly change the types of root exudates, and 15 compounds were identified and assumed to be potential biomarkers of Pb contamination.

Microbially mediated redox reactions also have profound effect on transformation of HM to less or non-toxic forms (Amstaetter et al., 2010). Outer membrane c-type cytochromes (OM c-Cyts), transouter membrane porin–cytochrome protein complex (Pcc), or MtrABC extracellular electron conduit play key role in microbial metal reduction processes (Shi et al., 2016). Such systems are well investigated in *Shewanella* and *Geobacter* species. Furthermore, HM may also be oxidized by specific enzymes. For example, multicopper oxidases such as CueO or CuiD and/or CopR are essentially required in Cu efflux. Whereas ChrA of chromate reductase perform reduction of Cr^+6^ to Cr^+3^. For Hg, the protein MerA reduces Hg^2+^ to lesser toxic Hg^0^. These proteins are up-regulated under toxic HM stress. There are several instances where HMT bacteria provide substantial aid in detoxification of HM in plants. A study of Chatterjee et al. (2009) on Cr-tolerant bacteria *Cellulosimicrobium cellulans* showed transformation of toxic Cr^6+^ to non-toxic Cr^3+^ and also its enhanced uptake in the shoot and root of green chili. Majumder et al. (2013) reported biotransformation of toxic As^3+^ to less toxic As^5+^ by As-oxidizing bacteria *Bacillus* sp. and *Geobacillus* sp. isolated from As-contaminated soils.

Bioaccumulation is also largely responsible for HM uptake and further detoxification by HMT-PGP microbes. There are two combined processes which are responsible for bioaccumulation of HM. Passive uptake or “biosorption” is metabolism-independent accumulation of metals by living or inactive non-living biomass or biological materials, whereas “active uptake” occurs only in living cells, requires metabolism and energy for the transport of metals (Gutierrez-Corona et al., 2016). Biosorption may involve one or a combination of different processes including complexation, coordination, chelation, ion exchange, microprecipitation, and entrapment (Pokethitiyook and Poolpak, 2016). Cell wall and associated functional groups like –SH, –OH, and –COOH, and other biomolecules have affinity for HM that helps in the biosorption process. Metal binding also involves chelators and metal-binding peptides, such as PC (glutathione-derived peptides) and metallothioneins (MT). PC and MT are produced by rhizospheric bacteria and fungi as well as by plants in response to HM stress and may result in the deposition of HM in microbial or plant cells (Miransari, 2011). MT are cysteine-rich metal peptides with high affinity for Cd, Cu, and Hg metals (Ahemad, 2014). In a study Murthy et al. (2011) found an increase in the MT biosynthesis in *Bacillus cereus* when it was exposed to increased Pb concentrations. Similarly, Sharma et al. (2017) also showed role of MT assisted periplasmic Pb sequestration by HMT *Providencia vermicola* strain SJ2A. Detoxification of HM via MT biosynthesis is also very well studied in HMT fungi. However, expression of the MT-related genes and their production in the presence HM has gained more attention in members of mycorrhizal fungi (Lanfranco et al., 2002; Lanfranco, 2007; Hložková et al., 2016). After entering into the cell final step of HM detoxification involves their sequestration or compartmentalization into different subcellular organelles. In mycorrhizal fungi, mainly vacuolar compartmentation of HM is noticed. Vacuolar compartmentalization of Zn, Cu, and Cd was observed in extraradical mycelium of *Glomus intraradices* renamed as *Rhizophagus irregularis* (González-Guerrero et al., 2008). Similarly, Yao et al. (2014) also showed vacuolar accumulation of Cd in Cd-exposed extraradical mycelium of *R. irregularis* in symbiosis with clover.

Microbial communities in the rhizosphere also excrete extracellular polymeric substances (EPS) such as polysaccharides, glycoprotein, lipopolysaccharide, and soluble peptide which possess substantial quantity of anion functional groups and help to remove or recover metals from the rhizosphere through biosorption (Ayangbenro and Babalola, 2017). EPS production by certain PGP microbes induce biofilm formation in response to the exposure of toxic HM. Biofilm formation enhances tolerance of microbial cells by forming a protective sheath as well as transform toxic metal ions into non-toxic forms after adsorption. EPS produced by rhizobia and other PGP microbes with multiple number of anionic groups are reported to sequester several types of HM (Gupta and Diwan, 2017).

## Plant Growth Promotion and HM Removal from Soil by HMT-PGP Microbes

HMT-PGP microbes not only contribute in growth enhancement of host plant but also accelerate the removal of HM from contaminated soils. This may occur due to enhanced or balanced plant growth under HM stress or by increasing the bioavailability of metals for easy uptake by plants and microbial cells. In rhizospheric microbial communities, PGP traits such as release of extracellular enzymes, siderophores, phytohormones, solubilization of insoluble form of minerals (phosphate, Zn, and K), and fixation of nitrogen provide plant growth promotion and simultaneously reduce adverse effect of HM on plants health.

Abiotic stresses (including HM) induce the production of stress hormone ethylene in plants, leading to suppressed plant growth and reduced root proliferation. Enhanced plant growth under HM contamination by enzyme 1-aminocyclopropane-1-carboxylate (ACC) deaminase producing microorganisms has been widely reported (Zhang et al., 2011; Han et al., 2015). High concentration of ethylene adversely affects root growth and proliferation in HM contaminated soils, ACC deaminase regulates its concentration by metabolizing ethylene precursor ACC and helps in plant survival under stress conditions. Phytohormone, indole acetic acid (IAA) produced by HMT-PGP microorganisms also induce root elongation and development of lateral and adventitious roots to overcome HM toxicity and hence improve plant growth. IAA producing strain *B. subtilis* SJ-101 stimulated the growth of *Brassica juncea* in Ni-contaminated soil (Zaidi et al., 2006). Similarly, Zn, Cu, Ni, and Co tolerant IAA producing strains were found to induce rapid root elongation in *B. juncea* in Cd contaminated soil (Belimov et al., 2005). Besides IAA and ACC deaminase, phosphate solubilizers, siderophore producers, and nitrogen fixing HMT-PGP microbes also assist in plant growth and root development by enhanced nutrient availability as well as by changing bioavailability of HM (Wu et al., 2010; Gupta et al., 2014). Pinter et al. (2017) found that siderophore production, phosphate solubilization, and nitrogen fixation activities of As tolerant *B. licheniformis*, *Micrococcus luteus*, and *Pseudomonas fluorescens* increased the biomass of grapevine in the presence of high As concentration. Environmental adaptability of Cd, Pb, and Cu resistant bacteria from rhizospheric soil of *Boehmeria nivea* growing around mine refineries was evaluated by Jiang et al. (2017) and they showed rhizosphere bacteria belonging to genera *Pseudomonas*, *Cupriavidus*, *Bacillus*, and *Acinetobacter* showed tolerance to Cd, Pb, and Cu at high concentrations. A wide array of PGP traits of rhizobia including fixation of nitrogen, solubilization of insoluble minerals such as phosphate, phytohormones production, release of siderophores, production of ACC deaminase, and volatile compounds such as acetoin and 2, 3-butanediol make rhizobia very good candidates for detoxification of HM and carry out rhizo and phytoremediation along with their partner legumes (Hao et al., 2014; Rangel et al., 2017). Rhizobia because of their symbiotic nitrogen fixation ability are well known to enhance the yield of legumes in HM contaminated soils (Arora et al., 2010). AM fungi are also reported to enhance the growth of plants in HM contaminated soils. Ruscitti et al. (2017) tested effect of inoculation of AM fungi on pepper growth in response to increasing soil Cu concentrations and found total dry weight and the leaf area was higher in mycorrhizal plants.

In spite of great potential, HMT-PGP microbes and their relations with host plants under HM contaminated soils are very less understood and required to be explored further. Further research using latest biotechnological tools and field studies should be done to determine synergistic action of HMT-PGP microbes in enhancing the growth of plant and their mechanisms of mobilization, transformation, and detoxification of HM in contaminated soils.

## Applications and Future Challenges

Use of HMT-PGP microbes along with their host plants for remediation of HM contaminated soils can be an eco-friendly and economic approach. However, there is still lack of knowledge to implement this technology at commercialization level. Accumulation of HM in plant tissues often downturns the remediation process when the contaminated sites are heavily polluted (Ma et al., 2011). In some instances where soil is contaminated with multiple types of HM, use of HMT-PGP microbes with additives (nutrients) is found to be more useful. Recently in microcosm-scale phytoextraction experiments, Franchi et al. (2017) showed that addition of thiosulfate with HMT-PGP microbes enhanced mobilization and uptake of As and Hg in *B. juncea* and *L. albus* grown in soil polluted with both metals. For phytoextraction of HM, use of non-food crops such as those used in timber or other commercial purposes (not involving human or animal consumption) can be targeted. This will result in removal of HM from the soil and non-transfer to the food chain. Use of genetically engineered microbes (GEM) well adapted to local conditions (soil and climatic) can also be done for efficient removal of HM from contaminated soils (Das et al., 2016; Gupta and Singh, 2017). Biostimulation of local microbial population by adding nutrients can also be an approach to encourage remediation and detoxification of HM contaminated soils (Fulekar et al., 2012). HMT microbes in consortium have recently been evaluated for their effectiveness in remediating HM from contaminated sites. A recent study by Migahed et al. (2017) showed that mixture of HMT bacterial biomass and fungal spores successfully removed Cr and Fe ions from industrial effluents. Entomopathogenic fungi can also be used in HM removal from contaminated soils (Gola et al., 2016). This can serve the purpose of biocontrol and remediation simultaneously in infested and polluted soils.

Pathway-engineering techniques to design or modify microbes and plants for enhanced HM removal can be useful (Mosa et al., 2016). Overexpression of metal-binding proteins, chelators, metal transforming, and detoxifying enzymes are the key traits being used in transgenic plants and GEM for remediating HM. Use of genetic engineering to construct “microbial biosensors” with enhanced potential of rapid detection of contaminated sites and accurate measurement of degree of contamination is also a promising technology (Dixit et al., 2015). Although GEM undoubtedly have greater remediation potential but studies related to their impact on eco-systems and regulation hurdles (related to biosecurity, diversity, end-users, government clearance) need to be overcome before the commercial use. Recent research shows that studies of plant microbiome from contaminated soils may boost existing phytoremediation technology for remediation of HM (Thijs et al., 2017).

## Conclusion

Contamination of agricultural soils with HM is becoming a serious environmental issue and finding economical and eco-friendly techniques to tackle this problem is on priority. Application of plant–microbe synergy to restore lands, contaminated with pollutants is a promising technique that is still in benign stage. Benefits of HMT-PGP microorganisms are immense as they perform multiple functions such as improved soil quality, enhanced plant growth, detoxification, and removal of HM from soil. However, further research is required to develop suitable bioformulations using HMT-PGP microbes for remediation and utilization of contaminated soils.

## Author Contributions

NA and JM conceived the idea; NA, RS, and JM prepared the manuscript; JM prepared illustration; and NA supervised the whole study.

## Conflict of Interest Statement

The authors declare that the research was conducted in the absence of any commercial or financial relationships that could be construed as a potential conflict of interest.
